# Supplementary material for: Barriers and Facilitators to Artificial Intelligence Implementation in Diabetes Management from Healthcare Workers’ Perspective: A Scoping Review
Source: Medicina (Kaunas). 2025 Aug 1;61(8):1403. doi: 10.3390/medicina61081403 (PMC12387811; doi:10.3390/medicina61081403)
Supplement: Supplementary file 1 [file medicina-61-01403-s001.zip › medicina-3757152-supplementary.pdf]

Barriers and Facilitators to Artificial Intelligence Implementation in Diabetes Management from Healthcare Workers' perspective: A Scoping Review.

Supplementary material

Table S1. PRISMA-ScR Checklist

Section and Topic	Item	Checklist item	Page
**Title**			
Title	1	Identify the report as a scoping review.	1
**Abstract**			
Structured summary	2	Provide a structured summary that includes (as applicable): background, objectives, eligibility criteria, sources of evidence, charting methods, results, and conclusions that relate to the review questions and objectives.	1-2
**Introduction**			
Rationale	3	Describe the rationale for the review in the context of what is already known. Explain why the review questions/objectives lend themselves to a scoping review approach.	2-3
Objectives	4	Provide an explicit statement of the questions and objectives being addressed with reference to their key elements (e.g., population or participants, concepts, and context) or other relevant key elements used to conceptualize the review questions and/or objectives.	3
**Methods**			
Protocol and registration	5	Indicate whether a review protocol exists; state if and where it can be accessed (e.g., a Web address); and if available, provide registration information, including the registration number.	3
Eligibility criteria	6	Specify characteristics of the sources of evidence used as eligibility criteria (e.g., years considered, language, and publication status), and provide a rationale.	4
Information sources	7	Describe all information sources in the search (e.g., databases with dates of coverage and contact with authors to identify additional sources), as well as the date the most recent search was executed.	3
Search	8	Present the full electronic search strategy for at least 1 database, including any limits used, such that it could be repeated.	3
Selection of sources of evidence†	9	State the process for selecting sources of evidence (i.e., screening and eligibility) included in the scoping review.	4
Data charting process	10	Describe the methods of charting data from the included sources of evidence (e.g., calibrated forms or forms that have been tested by the team before their use, and whether data charting was done independently or in duplicate) and any processes for obtaining and confirming data from investigators.	4-5
Data items	11	List and define all variables for which data were sought and any assumptions and simplifications made.	4-5
Critical appraisal of individual sources of evidence	12	If done, provide a rationale for conducting a critical appraisal of included sources of evidence; describe the methods used and how this information was used in any data synthesis (if appropriate).	4-5
Synthesis of results	13	Describe the methods of handling and summarizing the data that were charted.	5-6
**Results**			
Selection of sources of evidence	14	Give numbers of sources of evidence screened, assessed for eligibility, and included in the review, with reasons for exclusions at each stage, ideally using a flow diagram.	6
Characteristics of sources of evidence	15	For each source of evidence, present characteristics for which data were charted and provide the citations.	6-7
Critical appraisal within sources of evidence	16	If done, present data on critical appraisal of included sources of evidence (see item 12).	7
Results of individual sources of evidence	17	For each included source of evidence, present the relevant data that were charted that relate to the review questions and objectives.	7
Synthesis of results	18	Summarize and/or present the charting results as they relate to the review questions and objectives.	7-9
**Discussion**			
Summary of evidence	19	Summarize the main results (including an overview of concepts, themes, and types of evidence available), link to the review questions and objectives, and consider the relevance to key groups.	10-14
Limitations	20	Discuss the limitations of the scoping review process.	13
Conclusions	21	Provide a general interpretation of the results with respect to the review questions and objectives, as well as potential implications and/or next steps.	14
**Funding**			
Funding	22	Describe sources of funding for the included sources of evidence, as well as sources of funding for the scoping review. Describe the role of the funders of the scoping review.	15

Table S2. Search strategy

Database (results)	Search string
Scopus (1,757)	TITLE-ABS-KEY(("Artificial Intelligence" OR "AI" OR "Machine Learning" OR "ML" OR "Machine Learning Algorithms" OR "Extreme Learning Machines") AND ("Diabetes Complications" OR "Diabetes Mellitus, Type 2" OR "Diabetes Mellitus" OR "Diabetes, Gestational" OR "Diabete*" OR "Diabetes Mellitus, Type 1" OR "T2DM" OR "T2D" OR "T1DM" OR "T1D") AND ("Physician*" OR "Healthcare personnel" OR "healthcare worker*" OR "worker* in healthcare" OR "Nurses" OR "Nurse Practitioners" OR "General Practitioners" OR "Health Care Facilities Workforce and Services" OR "practitioner*" OR "HCW" OR "Skilled Nursing Facilities"))
PubMed (1,318)	("Artificial Intelligence"[Mesh] OR “AI” OR "Machine Learning"[Mesh] OR “ML” OR "Machine Learning Algorithms"[Mesh] OR "Extreme Learning Machines"[Mesh]) AND ("Diabetes Complications"[Mesh] OR "Diabetes Mellitus, Type 2"[Mesh] OR "Diabetes Mellitus"[Mesh] OR "Diabetes, Gestational"[Mesh] OR “Diabete*” OR "Diabetes Mellitus, Type 1"[Mesh] OR “T2DM” OR “T2D” OR “T1DM” OR “T1D”) AND ("Physicians"[Mesh] OR “Healthcare personnel”[Mesh] OR “healthcare worker*” OR “worker* in healthcare” OR “Nurses”[Mesh] OR "Nurse Practitioners"[Mesh] OR "General Practitioners"[Mesh] OR "Health Care Facilities Workforce and Services"[Mesh] OR “practitioner*” OR “HCW” OR "Skilled Nursing Facilities"[Mesh])
Embase (345)	("Artificial Intelligence" OR "AI" OR "Machine Learning" OR "ML" OR "Machine Learning Algorithms" OR "Extreme Learning Machines") AND ("Diabetes Complications" OR "Diabetes Mellitus, Type 2" OR "Diabetes Mellitus" OR "Diabetes, Gestational" OR "Diabete*" OR "Diabetes Mellitus, Type 1" OR "T2DM" OR "T2D" OR "T1DM" OR "T1D") AND ("Physician*" OR "Healthcare personnel" OR "healthcare worker*" OR "worker* in healthcare" OR "Nurses" OR "Nurse Practitioners" OR "General Practitioners" OR "Health Care Facilities Workforce and Services" OR "practitioner*" OR "HCW" OR "Skilled Nursing Facilities")
CINAHL (31)	Nurse OR Nursing OR (Nurse OR Nurses OR Nursing) OR Nurse Practitioners OR Nurse Specialist OR (Nurse Specialist OR CLinical Nurse Specialist) AND Diabetes OR Diabetes type 2 OR (Diabetes Type 2 OR Diabetes Mellitus Type 2 OR Diabetes) OR Diabetes type 1 AND Artificial Intelligence OR Artificial Intelligence in Healthcare OR (Artificial Intelligence OR AI OR A.I. OR Machine Learning OR Deep Learning)

Table S3. Cross-sectional studies quality appraisal

Study	1	2	3	4	5	6	7	8	Overall
Roy et al., 2024	No	No	Yes	Yes	Unclear	Yes	Yes	Yes	Low
Wewetzer et al., 2023	Yes	Yes	Yes	Yes	Unclear	No	Yes	Yes	Medium

Questions

1. Were the criteria for inclusion in the sample clearly defined?
2. Were the study subjects and the setting described in detail?
3. Was the exposure measured in a valid and reliable way?
4. Were objective, standard criteria used for measurement of the condition?
5. Were confounding factors identified?
6. Were strategies to deal with confounding factors stated?
7. Were the outcomes measured in a valid and reliable way?
8. Was appropriate statistical analysis used?

Reference Joanna Briggs Institute. Checklist for Analytical Cross Sectional Studies. 2020.

Table S4. Qualitative studies quality appraisal

Study	1	2	3	4	5	6	7	8	9	10	Overall
Liao et al., 2024	Yes		Yes	Yes	Yes	Yes	Yes	Yes	Yes	Yes	Excellent
Wahlich et al., 2024	Unclear	Yes	Yes	Yes	Yes	No	Unclear	Yes	Yes	Yes	Medium
Petersen et al., 2024	Yes	Yes	Yes	Yes	Yes	Yes	No	Yes	Yes	Yes	High
Held et al., 2022	No	Yes	Yes	Yes	Yes	Unclear	No	Yes	Yes	Yes	Medium

Questions

1. Congruity between the stated philosophical perspective and the research methodology
2. Congruity between the research methodology and the research question or objectives
3. Congruity between the research methodology and the methods used to collect data
4. Congruity between the research methodology and the representation and analysis of data
5. There is congruence between the research methodology and the interpretation of results
6. Locating the researcher culturally or theoretically
7. Influence of the researcher on the research, and vice-versa, is addressed
8. Representation of participants and their voices
9. Ethical approval by an appropriate body
10. Relationship of conclusions to analysis, or interpretation of the data

Reference Lockwood, C.; Munn, Z.; Porritt, K. Qualitative Research Synthesis. *International Journal of Evidence-Based Healthcare* **2015,** 13, 179–187.

Table S5. Mixed-methods studies quality appraisal

Study	S1	S2	1.1	1.2	1.3	1.4	1.5	2.1	2.2	2.3	2.4	2.5	3.1	3.2	3.3	3.4	3.5	4.1	4.2	4.3	4.4	4.5	5.1	5.2	5.3	5.4	5.5	Overall
Romero et al., 2019	Yes	Yes	-	-	-	-	-	-	-	-	-	-	Yes	Yes	Yes	No	Yes	-	-	-	-	-	-	-	-	-	-	High

Questions

Screening Questions

S1. Are there clear research questions?
S2. Do the collected data allow to address the research questions?

1. Qualitative
   1.1. Is the qualitative approach appropriate to answer the research question?
   1.2. Are the qualitative data collection methods adequate to address the research question?
   1.3. Are the findings adequately derived from the data?
   1.4. Is the interpretation of results sufficiently substantiated by data?
   1.5. Is there coherence between qualitative data sources, collection, analysis, and interpretation?
2. Quantitative randomized controlled trials
   2.1. Is randomization appropriately performed?
   2.2. Are the groups comparable at baseline?
   2.3. Are there complete outcome data?
   2.4. Are outcome assessors blinded to the intervention provided?
   2.5. Did the participants adhere to the assigned intervention?
3. Quantitative non-randomized
   3.1. Are the participants representative of the target population?
   3.2. Are measurements appropriate regarding both the outcome and intervention (or exposure)?
   3.3. Are there complete outcome data?
   3.4. Are the confounders accounted for in the design and analysis?
   3.5. During the study period, is the intervention administered (or exposure occurred) as intended?
4. Quantitative descriptive
   4.1. Is the sampling strategy relevant to address the research question?
   4.2. Is the sample representative of the target population?
   4.3. Are the measurements appropriate?
   4.4. Is the risk of nonresponse bias low?
   4.5. Is the statistical analysis appropriate to answer the research question?
5. Mixed methods
   5.1. Is there an adequate rationale for using a mixed methods design to address the research question?
   5.2. Are the different components of the study effectively integrated to answer the research question?
   5.3. Are the outputs of the integration of qualitative and quantitative components adequately interpreted?
   5.4. Are divergences and inconsistencies between quantitative and qualitative results adequately addressed?
   5.5. Do the different components of the study adhere to the quality criteria of each tradition of the methods involved?

Reference Hong, Q.N.; Fàbregues, S.; Bartlett, G.; Boardman, F.; Cargo, M.; Dagenais, P.; Gagnon, M.-P.; Griffiths, F.; Nicolau, B.; O'Cathain, A.; et al. The Mixed Methods Appraisal Tool (MMAT) Version 2018 for Information Professionals and Researchers. *EFI* **2018,** 34, 285–291.

Table S6. Complete data extraction

Study	Type of AI	Outcomes	Identified barriers	Identified facilitators
Liao et al., 2024	AI-assisted diagnosis system for diabetic retinopathy screening	CFIR domains (innovation; outer setting; inner setting; individual charachteristics; implementation process).	Innovation: unsatisfactory clinical performance, lack of adaptability for generated report, financial burden of AI software. Outer setting: lack of a collaborative network between primary/secondary and tertiary hospitals, lack of information security measures and certification, lack of collaboration between specialised and non-specialised clinical department. Inner setting: suboptimal data quality, misalignment between software functions and goals of healthcare institutions, lack of necessary medical supplies, lack of adequate training. Individual characteristics: unmet clinical needs, incompetence in understanding. Implementation process: AI reasoning mechanism, lack of feedback incorporation.	Innovation: improved clinical efficiency, strong empirical evidence of effectiveness, AI software trialability, easiness of use. Outer setting: National guidelines related to AI, deployment of AI software in peer hospitals. Inner setting: integration of AI software into existing hospital systems, regular communication channels within department. Individual characteristics: engagement of department head, engagement of hospital administrator. Implementation process: Involvement of clinicians.
Held et al, 2022	Smartphone-based and AI-supported diagnosis tools for the screening of diabetic rethinopathy	Attitudes towards implementation.	Negative personal attitude (suspicion that there is a lot of commerce behind AI projects, scepticism about the quality of the result by an AI, the fact that one could not fully understand how AI arrived at its result, danger of relying too much on AI, competencies that actually belong to this profession are at risk, duration of the examination and latency to the test result), influence on profession (potential tensions between GPs and established ophthalmologists due to potentially lower referral rates), others (how to deal with legal liability, data security concerns).	Positive Personal attitude (openness towards new procedures), organisation (creation of established treatment pathways between general practitioners and ophthalmologists), time (duration of the examination and latency to the test result), financial factors (appropriate remuneration, low acquisition costs, low running costs and the simplicity of billing), technical requirement (practical and user-friendly screening device, quick patient data input into the diagnostic software, simple installation and integration,ability to recognize more than one disease), patient welfare (strengthening of the primary doctor's filter function and an associated closer relationship between patient and GP), others (high instrument validity).
Petersen et al., 2024	AI for diabetes retinopathy screenig	Diabetes retiniophaty screening in general practice and use of AI.	Worsen both the patient experience and examination, patients would be unable to ask questions specific to their eyes, the GPs were cautiously in favour of the idea, concerns related to the financial aspect, validity concern regarding AI, may miss some retinopathy changes, worse examination, professional judgement should not be disregarded, clinician forgets to see the patient with AI, patient miss the opportunity to ask questions to the ophthalmologist, trust issue in AI, degrade the examination just using camera.	High quality of the images should be high and the task should not take doctor time. If screening were to be outsourced to their clinic, it would be beneficial to have AI can be a supporting tool. Easy to train a nurse for that examination, because it is carried by the camera. Perform the examination outside the ey clinic
Roy et al., India	AI-based diabetes diagnostic interventions	Behavioral intention to use AI; perceived usefulness; perceived ease of use; subjective norms; perceived risk; moderation by prior AI experience.	Perceived risk related to AI use (e.g., data security, diagnostic errors, liability concerns), lack of hands-on experience with AI tools, limited knowledge and training among physicians, fear of job displacement or changes in clinical autonomy, complexity of integrating AI into existing workflows, uncertainty about accuracy and trustworthiness of AI outputs.	Perceived usefulness of AI tools in improving diagnostic accuracy and efficiency, perceived ease of use supporting workflow integration, positive influence of subjective norms (peer and superior endorsement), prior experience with AI positively moderates acceptance, awareness of AI's potential to enhance early detection and diabetes management.
Romero et al., 2019	AI-powered clinical decision support system for identifying diabetes patients at risk of poor glycemic control	Staff perceptions of care coordination and patient preparation; familiarity and excitement about AI; satisfaction with CDS tool; willingness to recommend/use the tool.	Poorly tailored or irrelevant recommendations by the AI system, high false-positive rate in patient risk classification, lack of integration with existing electronic health record systems (EHR interoperability issues), increased workload due to extra steps outside routine workflow, low provider confidence and engagement with the tool post-use, difficulty understanding how recommendations were generated ("black box" problem)	The AI tool prompted valuable team dialogue around high-risk patients' needs. Helped improve perception of care coordination across the care team. Increased staff familiarity with how AI works in real clinical settings. Identified high-risk patients effectively, triggering proactive review even when recommendations weren't adopted directly

Study	Type of AI	Outcomes	Identified barriers	Identified facilitators
Wewetzer et al., 2023	AI-assisted technology for diabetic rethinopathy	Determinants of AI implementation.	Financial factors: acquisition and operating costs: (physicians expressed concerns about the initial costs of purchasing AI devices and the ongoing operating expenses associated with their use), insufficient compensation (the lack of clear and adequate reimbursement policies for AI-based screening is a significant barrier to adoption of these technologies), technical integration, compatibility with existing systems (difficulties in integrating AI devices into existing medical practice information systems can hinder effective implementation), software installation (challenges with installing and configuring the software required to operate AI devices can be a technical barrier), clinical concerns, diagnostic limitations of AI (hysicians expressed concerns that AI may not detect other eye conditions besides DR, leading to incomplete diagnoses), reliability of AI diagnoses: (doubts about the accuracy and reliability of diagnoses provided by AI may negatively impact physicians' confidence in such tools).	Positive attitudes towards AI: perception of AI as a support: (doctors see AI not as a threat, but as a tool that can improve the efficiency and quality of care provided), modernization of medical practice (the adoption of AI technologies is perceived as a sign of modernity and can improve the image of medical practice in the eyes of patients). Benefits for patients: timely and accessible screening (implementing AI screening directly in medical offices can facilitate patients' access to early diagnoses, reducing waiting times for specialist visits), improved quality of care (the use of AI can contribute to more effective management of DR, improving clinical outcomes for patients). Support for medical staff: delegability of tasks (the possibility of delegating some tasks related to AI screening to non-medical staff can relieve the workload of doctors and improve operational efficiency). Training and professional development: the adoption of AI provides training and professional development opportunities for medical and non-medical personnel, promoting an environment of continuous learning.
Wahlich et al., 2024	AI-assisted diabetic eye screening	Thoughts towards AI technology (i.e., data regulation, security, responsibility, and governance; trust; human involvement, screening experience, and patient and practitioner relationship; practicalities of AI implementation; efficiency; inequalities; impact on societal changes).	Thoughts towards AI technology: AI could not detect individual retinal disease, AI has limited use, AI could be problematic in the future, Lack of detail on AI technology, Missing detection of other conditions, Negative opinion of AI. More information needed: Lack of knowledge in subject area, Misunderstanding around AI, More education for patients, More research required, Need for more information about the potential of AI, Regular updates with research. Impact on grading workload: Impact on job recruitment, skilss, staff morale, job security concern, Staff deployment to other roles, Training requirements for correct use. Data regulation, security, responsibility, and governance: Data security concerns, Human accountability required, Importance of quality assurance, Meeting required standardRegular updates with technology required, Training of AI systems. Trust: Concerns over errors with AI, Greater patient reassurance about AI, Impact on patient confidence + trustcurrent lack of confidence in AI, Need more information before trust established. Human involvement, screening experience, and patient and practitioner relationship: balance between AI and human, concern for human future, human contact remain fundamental, human oversight in AI-driven processes, human are superior. Practicalities of AI implementation: HCP were more sceptical about the financial benefits, pointing out the high costs associated with implementation, maintenance, and security of AI systems. They worried that AI might not provide good value for money. HCP also raised concerns that AI might not necessarily enhance patient capacity but instead lead to budgetary savings at the expense of service expansion. Inequalities: biases in AI algorithms, particularly regarding ethnicity. Biases in AI technology by ethnicity, Implications on patient attendance. Accuracy and reliability of AI technology: Concern over accuracy and reliability of AI. Ensuring human-comparable accuracy and reliability, extensive testing and validation before AI can be fully trusted. Impact on societal change: Negative media coverage around AI, Misleading media information.	Thoughts towards AI technology: Accepting of change, AI is the future, AI technology could be a great tool, benefit of AI technology if implemented correctly, High confidence in AI technology, long-term benefits, AI should have been implemented earlier. AI technology is a powerful tool. Future (development) of AI technology, Inevitability of AI in the future, Use of AI natural progression in diabetic eye screening. Impact on grading workload: Positive impact on workflow. Efficiency: potential for AI to enhance efficiency. They agreed that AI could speed up processes, particularly in screening and grading tasks. AI's capacity to efficiently manage large volumes of data, potentially improving overall workflow and resource allocation. They envisioned AI as a way to free up human resources for other critical tasks within the department. Accuracy and reliability of AI technology. Reduce human errors caused by fatigue and repetitive tasks.
